# Machine Learning-Based Identification of Biomarkers for Early-Stage Non-Small Cell Lung Cancer Through Gene Expression Analysis

**DOI:** 10.3390/ijms27104282

**Published:** 2026-05-11

**Authors:** Zorka Szollár, Fanni Dzsubák, Ádám Ürmös, Barbara N. Borsos, Balázs Bende, Zoltán G. Páhi, Tibor Pankotai

**Affiliations:** 1 Hungarian Centre of Excellence for Molecular Medicine (HCEMM), Genome Integrity and DNA Repair Core Group, 6728 Szeged, Hungary; szollarzorka@gmail.com (Z.S.); dzsubak.fanni@gmail.com (F.D.); adam.urmos@hcemm.eu (Á.Ü.); barbara.borsos@hcemm.eu (B.N.B.); balazs.bende@hcemm.eu (B.B.); 2 Competence Centre of the Life Sciences Cluster of the Centre of Excellence for Interdisciplinary Research, Development and Innovation, University of Szeged, 6720 Szeged, Hungary

**Keywords:** non-small cell lung cancer, gene expression, RNA-sequencing, bioinformatic analysis, biomarkers

## Abstract

Tumor progression is primarily driven by DNA mutations; however, this mechanism alone does not fully account for all aspects of tumor development. Beyond genetic alterations, epigenetic changes also significantly influence the mutational landscape and affect gene expression without altering the DNA sequence. To gain a more comprehensive understanding of these regulatory mechanisms, it is essential to analyze gene expression at the transcriptional level. In this study, we examined non-small cell lung cancer (NSCLC) samples to identify specific gene expression changes, particularly in early-stage tumors. We conducted a bioinformatic analysis of RNA-sequencing data, followed by validation using an independent dataset from The Cancer Genome Atlas. Our analysis revealed a set of differentially expressed genes, seven of which were validated in patient-derived samples. Among these genes, *EFNA4* and *TEDC2* were significantly upregulated, whereas *CDC42EP2*, *STX11*, *THBD*, *TMEM88*, and *GPM6A* were notably downregulated in tumor tissues compared with adjacent normal tissues. Our findings highlight a distinct gene expression signature that differentiates NSCLC samples from normal lung tissues at the transcriptional level. These results underscore the potential of transcriptomic profiling as a promising tool for early-stage cancer detection and biomarker discovery.

## 1. Introduction

Lung cancer is the leading cause of cancer-related deaths worldwide, with non-small cell lung cancer (NSCLC) accounting for 85% of cases [1]. Despite available interventions such as surgery or therapies, including radiotherapy, chemotherapy, and targeted therapy, the prognosis for many patients remains poor, with a five-year survival rate of only 23% [2]. Many early-stage patients who undergo surgery still face risks of recurrence or metastases, which often necessitate systemic treatment [3].

Advancements in DNA-based diagnostics have improved cancer treatment by focusing on mutation detection, though this represents only part of the disease’s complexity [4,5]. Testing for early and locally advanced NSCLC includes blood tests, medical imaging, pulmonary function tests, and biomarker testing. These evaluations often assess programmed death-ligand 1 (*PD-L1*) levels, epidermal growth factor receptor (*EGFR*) mutations, and anaplastic lymphoma kinase (*ALK*) rearrangements [6].

The National Comprehensive Cancer Network guidelines recommend broad-panel next-generation sequencing (NGS) as the primary method for identifying mutations associated with NSCLC. If no driver oncogene mutations are detected, RNA sequencing (RNA-seq) can be used to identify gene fusions [7]. *EGFR* mutations and *ALK* rearrangements can be detected with polymerase chain reaction (PCR) or NGS and fluorescence in situ hybridization or immunohistochemistry, respectively [8,9]. Besides *EGFR* and *ALK* rearrangements, NSCLC is driven by diverse mutations, each affecting prognosis and treatment. Among these, *TP53* and Kirsten rat sarcoma viral oncogene homolog (*KRAS*) mutations are associated with poor survival, whereas *EGFR* alterations are amenable to multiple targeted therapies [10,11]. Additionally, *ALK* and ROS proto-oncogene 1, receptor tyrosine kinase (*ROS1*) rearrangements primarily occur in younger, non-smoking patients; however, the available inhibitors often face resistance [12]. The amplification of the MET proto-oncogene, receptor tyrosine kinase (*MET*), which is linked to aggressive behavior, can be treated with specific drugs [13]. Phosphatidylinositol-4,5-bisphosphate 3-kinase catalytic subunit alpha (*PIK3CA*) mutations are common in squamous carcinoma. B-Raf proto-oncogene, serine/threonine kinase (*BRAF*) V600E and human epidermal growth factor receptor 2 (*HER2*) alterations, though subtle, have significant clinical implications [14,15].

Lung cancer progression is driven by genetic mutations and/or epigenetic modifications, leading to intratumoral heterogeneity by promoting lineage plasticity. Epigenetic dysregulation contributes to tumor diversity and resistance to targeted therapies [16]. These alterations play a critical role in hallmark cancer processes, including uncontrolled proliferation, resistance to apoptosis, angiogenesis, and metastasis [17]. Lung tumors often exhibit global DNA hypomethylation, leading to genomic instability, along with hypermethylation of CpG islands, which silences tumor suppressor genes and promotes oncogenesis; consequently, this manifests in transcriptomic dysregulation [18]. Epigenetic changes significantly impact transcription activity and gene expression. RNA analysis may reveal these shifts in expression, providing insight into how epigenetic modifications affect cellular function and therapeutic response [19]. While tissue biopsies remain the gold standard for genotyping, plasma-based circulating tumor DNA (ctDNA) liquid biopsies complement tissue testing, providing a more comprehensive analysis [20,21]. Standard diagnostics and therapies offer essential benefits but also face limitations. By incorporating RNA analysis and liquid biopsies for ctDNA analysis, a minimally invasive alternative to traditional tissue biopsies can be developed. Integrating ctDNA analysis with multi-omics approaches holds promise for improved cancer management [21,22].

In this study, we investigate the role of transcriptomic changes in NSCLC, with a particular focus on early-stage disease. By leveraging RNA-seq data and bioinformatics analysis, we have identified differentially expressed genes (DEGs) that can serve as potential biomarkers for early detection of NSCLC. Our results have been validated using an independent dataset from The Cancer Genome Atlas (TCGA) and confirmed in patient samples with RT–qPCR analysis. Our findings reveal a distinct gene expression signature that distinguishes NSCLC from normal tissue, underscoring the importance of transcriptional profiling in understanding tumor development. Notably, we have identified seven genes with significant expression alterations: two upregulated genes—ephrin A4 (*EFNA4*) and tubulin epsilon and delta complex 2 (*TEDC2*)—and five downregulated genes—CDC42 effector protein 2 (*CDC42EP2*), syntaxin 11 (*STX11*), thrombomodulin (*THBD*), transmembrane protein 88 (*TMEM88*), and glycoprotein M6A (*GPM6A*). These genes may play crucial roles in the progression of NSCLC and can serve as biomarkers for early-stage detection.

## 2. Results

**Figure 1:**
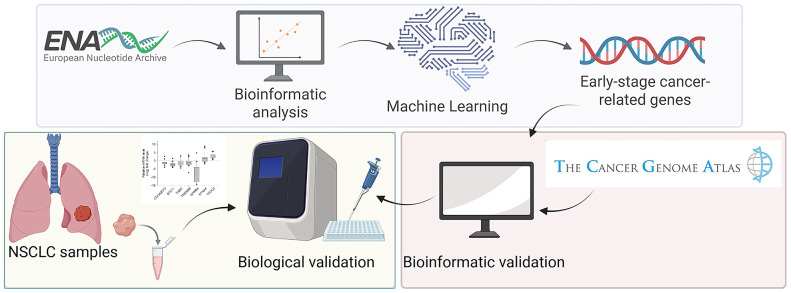
Schematic overview of the experimental design and analytical workflow.

Jeong-Sun Seo et al. conducted the first large-scale RNA-seq study of lung adenocarcinoma, providing valuable insights into its molecular profile. They comprehensively profiled lung adenocarcinoma across all stages, identifying key driver somatic mutations, such as *EGFR*, *KRAS*, neuroblastoma rat sarcoma viral oncogene (*NRAS*), *BRAF*, *PIK3CA*, *MET*, and catenin beta 1 (*CTNNB1*). Moreover, they discovered potential new drivers, including lemur tyrosine kinase 2 (*LMTK2*), AT-rich interacting domain 1A (*ARID1A*), neurogenic locus notch homolog protein 2 (*NOTCH2*), and SWI/SNF-related, matrix-associated, actin-dependent regulator of chromatin, subfamily a, member 4 (*SMARCA4*). In contrast, our study focused specifically on the transcriptomic profile during the early stages of this malignancy [23] (Figure 1).

**Figure 2:**
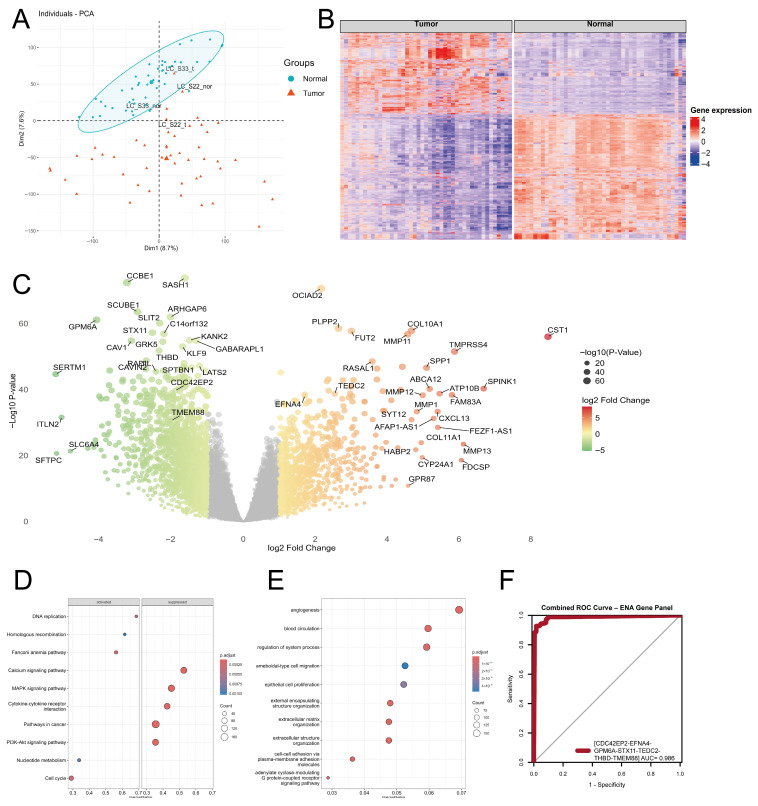
Multi-layered transcriptomic analysis of tumor versus normal samples. (**A**) Principal Component Analysis (PCA) plot illustrating the distribution of samples from the ENA dataset. Samples highlighted were excluded due to abnormal clustering; specifically, a tumor sample exhibited expression patterns that were too similar to those of normal tissues, while a normal sample did not sufficiently cluster with other normal controls. (**B**) Heatmap displaying the normalized expression levels (transcripts per million [TPM], log_2_-transformed) of significantly differentially expressed genes (DEGs) between tumor and normal samples. (**C**) Volcano plot depicting log_2_ fold change values of DEGs. Genes with log_2_ fold change beyond ±1 and adjusted *p*-value < 0.05 are highlighted, and the most significant DEGs are annotated with their gene names. (**D**) Dot plot displaying pathway enrichment results for the DEGs. Enrichment analysis was conducted using the clusterProfiler R package, with 10,000 permutations for increased accuracy. A fixed random seed was used to ensure reproducibility. (**E**) Dot plot showing the result of the over-representation analysis (ORA), which was conducted using enrichGO from clusterProfiler, applying adjusted *p*-value < 0.10 thresholds to identify significantly enriched terms. (**F**) Combined Receiver Operating Characteristic (ROC) analysis of the seven genes on the ENA dataset.

We analyzed transcriptomic data from 31 stage 1A and 24 stage 1B tumor samples, along with their matched adjacent normal pairs. All specimens were obtained as fresh surgical samples. Among the 30 male and 25 female participants, 27 individuals were smokers. Eight tumor samples lacked matched adjacent normal samples and were therefore excluded from this study. Principal Component Analysis (PCA) was performed to assess potential outliers among the samples (Figure 2A). During this preliminary quality control, two sample pairs were excluded due to their outlier nature.

Using RNA-seq data from samples that met the inclusion criteria, we utilized the edgeR package, which is suitable for paired RNA-seq experiments, to identify genes with altered expression between tumor and adjacent normal samples. Our differential gene expression analysis identified 3421 DEGs, out of 19,886 analyzed genes. Significance was defined by an adjusted *p*-value of <0.05 and an absolute log_2_ fold change > 1 (Figure 2B,C).

To confirm the biological relevance of these identified genes in relation to NSCLC, multiple pathway analyses, including the KEGG, GO, and Reactome databases, were performed. The GSEA using the KEGG database highlighted that the most significantly activated pathways were those associated with DNA replication, homologous recombination, and cell cycle (Figure 2D, Supplementary Figure S1A–E). These pathways are involved in various cellular processes such as maintaining genomic integrity, coordinating accurate DNA synthesis, and regulating checkpoint control. These processes are often disrupted in various tumors, leading to the development of targeted therapies that specifically inhibit proteins involved in these pathways. The most suppressed pathways are the calcium and the MAPK signaling pathways. The downregulation of these pathways contributes to the regulation of proliferation, differentiation, and apoptosis. Several targeted therapies have been developed to selectively inhibit the proteins involved in these suppressed circuits. Over-representation analysis (ORA) based on the GO database revealed significant over-representation of pathways associated with angiogenesis, extracellular matrix organization, and epithelial cell proliferation. Dysregulation of these processes promotes tumor progression by enhancing extracellular matrix remodeling, facilitating invasion and metastasis, and supporting neovascularization required for sustained tumor growth. (Figure 2E, Supplementary Figure S1F–H).

Transcripts per million (TPM) values were calculated for sample normalization. TPM was chosen for its ability to normalize both gene length and sequencing depth, enabling accurate comparisons across samples. Utilizing the 3424 genes that were significantly differentially expressed and their TPM values, we built a Random Forest model to identify a gene expression pattern that effectively distinguishes tumor samples from normal samples with high accuracy. Random Forest was selected for this purpose due to its superior performance compared to other supervised learning models we evaluated.

**Figure 3:**
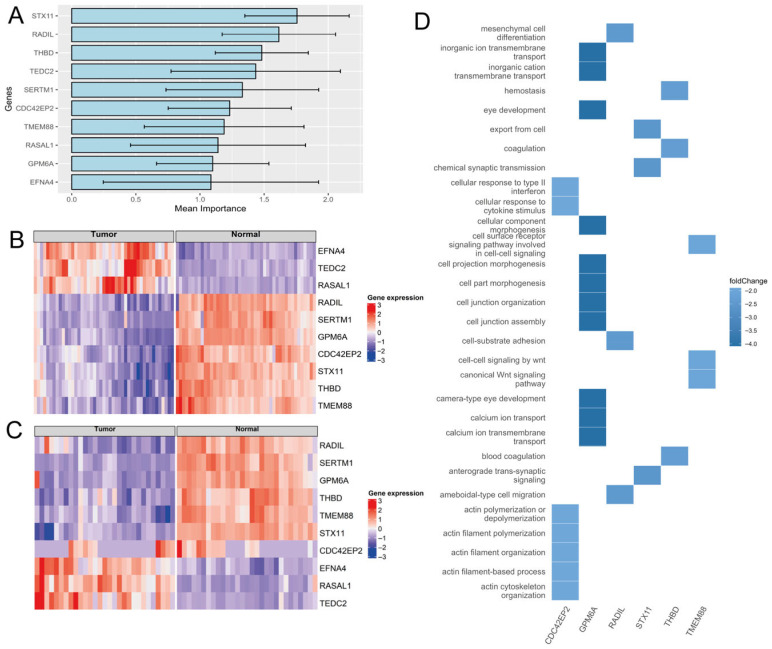
Validation and functional relevance of top classifier genes identified by Random Forest analysis. (**A**) Bar plot illustrating the top 10 genes with the highest importance scores as identified by the Random Forest classifier. (**B**) Heatmap displaying the normalized expression levels (TPM, log_2_-transformed) of the 10 most informative genes—*EFNA4*, *TEDC2*, *RASAL1*, *RADIL*, *SERTM1*, *GPM6A*, *CDC42EP2*, *STX11*, *THBD*, and *TMEM88*—in the ENA dataset. These genes were selected based on their classification importance in the Random Forest model. (**C**) Heatmap displaying the expression of these genes—*RADIL*, *SERTM1*, *GPM6A*, *THBD*, *TMEM88*, *STX11*, *EFNA4*, *RASAL1*, *CDC42EP2*, and *TEDC2*—in TCGA samples, which are also represented in the ENA dataset. The replication of expression patterns across both datasets supports the robustness of the classification model. Expression values were normalized using TPM and log_2_ transformation. (**D**) Heatmap summarizing the biological pathways most affected by the differential expression of the top 10 genes. Enriched pathways include those related to cytokine response, cell junction assembly, cell–cell signaling, and signaling receptor activity.

In a 10-iteration loop, we randomly split the dataset into training (80%) and testing (20%) sets. Furthermore, a 10-fold cross-validation was implemented. By running the model multiple times, we aimed to minimize the impact of randomization and enhance its generalizability. The optimal number of trees (ntree) was set to 200, which proved to be the best-performing configuration. The number of variables randomly selected at each node was determined using a common practice: the square root of 3424 and the total number of genes in the model. Across 10 iterations, the model achieved an average accuracy of 97.8%, indicating it can classify a sample from the testing set as a tumor or a normal sample with 97.8% accuracy. To identify the genes that contributed most to the classification, we used the importance function. From the ranked list of genes provided by this function, we selected the top 10 genes for further analysis: *STX11*, *RADIL*, *THBD*, *TEDC2*, *SERTM1*, *CDC42EP2*, *TMEM88*, *RASAL1*, *GPM6A*, and *EFNA4* (Figure 3A,B).

**Figure 4:**
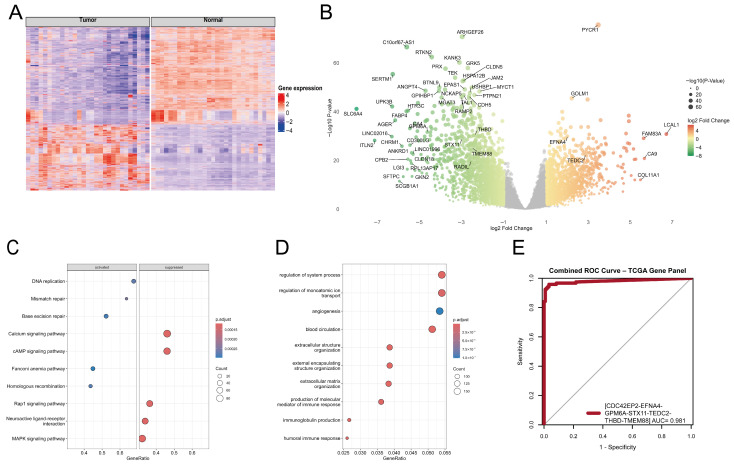
Multi-layered transcriptomic analysis of tumor versus normal samples in the TCGA dataset. (**A**) Heatmap displaying the normalized expression levels (TPM, log_2_-transformed) of significant DEGs in tumor samples compared with normal samples from the TCGA dataset. Significance was determined by an adjusted *p*-value of <0.05 and log_2_ fold change beyond ±1. (**B**) Volcano plot illustrating the log_2_ fold change values of DEGs from TCGA. Genes that exhibit a log_2_ fold change beyond ±1 and an adjusted *p*-value of <0.05 are highlighted, and the top DEGs are annotated. (**C**,**D**) Dot plots demonstrating GSEA and ORA for DEGs derived from TCGA. These analyses were performed using the clusterProfiler package with the parameters consistent with those in previous analyses (Figure 2D,E), ensuring both consistency and reproducibility. (**E**) Combined ROC analysis of the seven genes on the TCGA dataset.

To validate our results, we constructed an independent Random Forest model using the top 10 genes and their TPM values as the training set. We employed a TCGA dataset as a testing set to determine whether the gene expression patterns of these 10 genes were identified in an independent secondary dataset. By using the same model parameters as in the first model, this new model achieved an average accuracy of 95.52%, demonstrating strong performance in distinguishing tumors from normal samples (Figure 3C). The biological relevance of these 10 genes and the pathways in which they are involved are illustrated in Figure 3D. Collectively, this finding further confirms the potential of our gene expression pattern as a robust and reliable diagnostic marker for early-stage NSCLC. The high level of accordance between our internal dataset and the TCGA data strongly supports the validity and generalizability of our findings (Figure 4, Supplementary Figure S2).

To further validate our results, we performed RT–qPCR measurements on tumor and adjacent normal tissue samples from human subjects. From the genes identified in the NGS analysis, we selected 7 genes (i.e., *CDC42EP2*, *STX11*, *THBD*, *TMEM88*, *GPM6A*, *EFNA4*, and *TEDC2*) based on differential expression between normal and tumor samples, prioritizing those with the most significant expression changes. To ensure the robustness of our findings, we established a well-defined patient cohort, comprising 24 individuals diagnosed with NSCLC (Table 1). Frozen tissue samples were collected from both the tumor and adjacent normal tissues from the same patients. An expert pathologist evaluated the tumor samples to maintain sample integrity and ensure an accurate representation of tumor biology. Only samples with at least 30% tumor content were included in the analysis.

**Figure 5:**
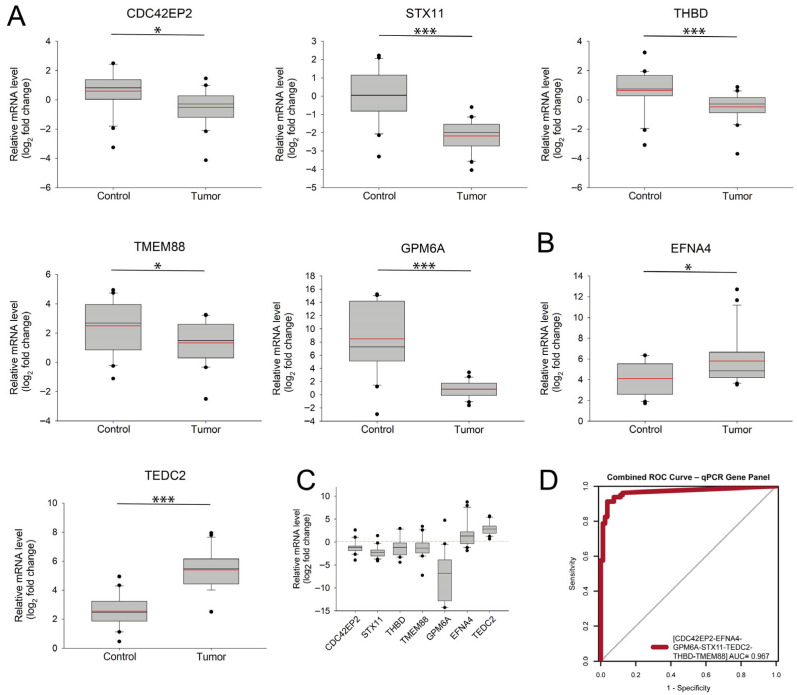
Validation of DEGs in NSCLC using RT–qPCR in a Hungarian patient cohort. RT–qPCR analysis was performed on tumor and adjacent normal tissue samples from 20 NSCLC patients to validate the differential expression of selected genes identified in the NGS dataset. Expression levels were log_2_-transformed for visualization. (**A**) Boxplot displaying the expression levels of downregulated genes in NSCLC tumor samples compared with matched normal tissues, including *CDC42EP2*, *STX11*, *THBD*, *TMEM88*, and *GPM6A*. Statistical significance was determined using the independent-samples *t*-test: * *p*-value < 0.05; *** *p*-value < 0.001. (**B**) Boxplot displaying the expression levels of upregulated genes *EFNA4* and *TEDC2*, which were significantly higher in tumor samples. Statistical significance was determined using the independent-samples *t*-test: * *p*-value < 0.05; *** *p*-value < 0.001. (**C**) Paired boxplot visualization comparing gene expression between tumor and matched normal samples on a per-patient basis, highlighting individual variation. The red line indicates the median expression level of the control group. (**D**) Combined ROC analysis of the 7-gene RT–qPCR panel.

We quantified RNA expression levels across these samples using RT–qPCR, calculated the mean expression values, and visualized the average expression levels (Figure 5). The RT–qPCR results demonstrated a strong correlation with the NGS data, reinforcing the reliability of our transcriptomic analysis. Specifically, five genes—*CDC42EP2*, *STX11*, *THBD*, *TMEM88*, and *GPM6A*—showed marked downregulation in tumor tissue compared with matched adjacent normal samples (Figure 5A). This downregulation may indicate a potential tumor suppressor function, as reduced expression levels can contribute to impaired cellular homeostasis, loss of cell adhesion, or enhanced metastatic potential (Figure 3D). In contrast, two genes, *EFNA4* and *TEDC2*, exhibited significantly elevated mRNA levels in tumor samples (Figure 5B), suggesting their potential role in tumor progression or oncogenic signaling pathways.

To assess the discrimination potential of the combined expression of seven genes (*CDC42EP2*, *EFNA4*, *GPM6A*, *STX11*, *TEDC2*, *THBD*, and *TMEM88*), we applied a generalized linear model (GLM)-based approach similar to that implemented in the *combiROC* R package. Our results show that the combined Receiver Operating Characteristic (ROC) curve derived from sequencing data achieved a higher area under the curve (AUC) than that from RT–qPCR data. Importantly, two independent validation datasets were used, including the RT–qPCR dataset, which serves as biological validation. Although differences in performance metrics across datasets were expected, the combined ROC analysis of seven genes consistently yielded a well-separated classification model across all three cohorts (i.e., ENA, TCGA, and RT–qPCR).

These findings align with existing literature, further supporting the involvement of these genes in lung cancer biology. The observed gene expression patterns across all three independent cohorts suggest that this gene set could serve as a potential diagnostic or prognostic biomarker panel for NSCLC. However, further functional validation, mechanistic exploration of these gene candidates, and assessment of their potential utility in clinical applications, such as patient stratification and therapeutic targeting, still need to be elucidated.

## 3. Discussion

Tumor progression is a highly complex process that primarily involves transcriptomic reprogramming in cells [16]. Epigenetic and transcriptomic changes are increasingly recognized as early indicators of tumor development. To explore whether tumor and normal tissues in early-stage NSCLC can be robustly distinguished based on these changes, we conducted integrated bioinformatic and experimental analyses. Our findings reveal a seven-gene expression signature that significantly differentiates early-stage NSCLC tumors from adjacent normal tissues. This signature was validated across three independent cohorts and confirmed with RT–qPCR in a Hungarian cohort, underscoring its reliability and translational potential. Recent studies further support the concept that transcriptomic dysregulation frequently precedes overt genomic instability during early lung tumorigenesis. While mutation-based diagnostics remain central in NSCLC management, increasing evidence indicates that epigenetic alterations, including DNA methylation changes, chromatin remodeling, and histone modification patterns, can drive transcriptional reprogramming at very early stages of malignant transformation. Baumann et al. have recently described these alterations as “epigenomic echoes,” emphasizing that early-stage NSCLC often exhibits stable transcriptomic disturbances even before the accumulation of dominant driver mutations [19]. Similarly, Marei highlighted that epigenetic regulators shape therapeutic resistance and lineage plasticity by maintaining aberrant transcriptional states rather than solely inducing DNA mutations [16].

This concept strongly supports our analytical strategy, which focuses on transcript-level alterations rather than predefined mutational panels. Unlike DNA-based testing that relies on detecting specific driver alterations such as *EGFR*, *ALK*, or *KRAS*, transcriptomic profiling captures the functional output of both genetic and epigenetic dysregulation. This process is particularly relevant in early-stage tumors, where mutational burden may remain low, and ctDNA sensitivity is limited. The observed dysregulation of genes involved in angiogenesis, extracellular matrix remodeling, and Wnt/β-catenin signaling in our dataset likely reflects these upstream epigenetic disturbances. Therefore, our seven-gene signature may represent not only diagnostic markers but also transcriptional readouts of early oncogenic reprogramming.

Our study offers several improvements over existing diagnostic modalities for early-stage NSCLC. Currently, low-dose CT screening remains the gold standard for early detection, yet it suffers from high false-positive rates and lacks molecular resolution [4]. Tissue- or plasma-based genomic profiling—such as testing for *EGFR* mutations, *ALK*/*ROS1* fusions, and *PD-L1* expression—is routinely employed for therapeutic guidance, but not for early detection. Although ctDNA is a promising minimally invasive tool, its efficacy is often limited in early-stage disease due to low tumor burden. In contrast, our RNA-seq-based transcriptomic profiling captures gene expression shifts that may occur before significant genomic instability and detectable mutations arise. This method provides a complementary and potentially more sensitive approach for early diagnosis. Importantly, our pipeline does not rely on predefined mutation panels; instead, it enables data-driven discovery of dysregulated gene signatures that reflect early malignant transformation.

To identify molecular features that distinguish tumors from normal tissue, we applied a machine learning-based feature selection pipeline. Using a Random Forest classifier, we ranked genes based on their contribution to classification accuracy. Subsequently, we validated the top candidates based on their biological relevance and expression patterns. Among the most informative DEGs, *EFNA4* and *TEDC2* were significantly upregulated in tumor tissues. *EFNA4*, a member of the ephrin family involved in cell signaling, has been linked to tumor progression and angiogenesis in various cancers, including lung cancer [24]. *EFNA4* upregulation may reflect enhanced tumor cell communication, migration, and neovascularization in early tumorigenesis, and it also promotes lymph node metastasis in later stages [24,25]. *TEDC2* has recently been shown to be implicated in cell cycle regulation, and higher expression levels have been associated with a worse prognosis [26]. On the other hand, *CDC42EP2*, *STX11*, *THBD*, *TMEM88*, and *GPM6A* were markedly downregulated in tumor samples. These genes have diverse biological roles, including vesicular transport (*STX11*) and immune signaling (*THBD*) [27,28]. Their suppression in early-stage tumors may indicate loss of normal tissue architecture, immune evasion, or altered differentiation pathways—hallmarks of malignant transformation.

Recent studies further support the importance of transcriptional regulation and epithelial remodeling as central mechanisms in early lung tumorigenesis. Beyond classical driver mutations, disruption of transcriptional control and RNA-processing pathways can promote epithelial–mesenchymal transition, immune escape, and metastatic competence through coordinated reprogramming of gene expression [29,30]. RNA-binding proteins have emerged as critical regulators of these processes. For example, recent work has demonstrated that RBMX functions as an important regulator of transcriptional stability and epithelial plasticity, contributing to tumor-associated signaling and progression in lung cancer models [31]. Similarly, studies investigating infection-associated lung disease have reported that host transcriptional regulators and RNA-binding proteins coordinate epithelial remodeling and inflammatory signaling, thereby providing mechanistic links between transcriptional dysregulation and disease progression [32]. These findings are highly relevant to our results, particularly for genes such as *TMEM88*, *CDC42EP2*, and *THBD*, whose downregulation may reflect disruption of epithelial integrity and altered signaling pathways rather than isolated gene-specific effects. Therefore, the identified seven-gene signature may represent a broader transcriptional reprogramming event associated with early malignant transformation rather than simply a collection of independent biomarkers. For instance, *TMEM88* is known to negatively regulate the Wnt/β-catenin signaling pathway, a key driver of NSCLC progression; its downregulation may facilitate tumorigenesis and metastasis [33]. Importantly, while some of these genes have been implicated in lung cancer, others—such as *TEDC2* and *CDC42EP2*—remain poorly characterized in this context, suggesting that our approach has revealed previously unrecognized components of early tumor biology.

Because of its exclusive focus on early-stage (i.e., stage 1) disease and its incorporation of machine learning-based feature selection across multiple datasets, our study complements prior RNA-seq efforts, such as the comprehensive lung adenocarcinoma transcriptome reported by Seo and colleagues [23]. While Seo’s dataset spanned all tumor stages and emphasized mutation discovery, our approach directly addresses the need for molecular stratification at early stages using gene expression as the central feature. Our Random Forest model achieved a classification accuracy of over 95% in both internal and TCGA-derived testing datasets, indicating the diagnostic discriminative capacity of the identified gene set. Furthermore, by validating these findings across ethnically diverse cohorts and Hungarian clinical samples, we support the generalizability of the signature.

From a translational perspective, the reproducibility of this gene set across populations with different nationalities suggests it may serve as a ubiquitous molecular marker, given the well-documented genomic variability of NSCLC worldwide. Previous studies have emphasized substantial heterogeneity across NSCLC subtypes and populations, with significant differences in the prevalence of driver mutations, gene fusions, and transcriptomic profiles—largely influenced by ancestry, sex, and smoking status [34,35]. Although the observed consistency of this transcriptomic pattern is promising, the broader applicability requires larger, prospective validation. Moreover, transcriptomic consistency often correlates with core regulatory functions, suggesting that these genes might also serve as early-stage therapeutic targets, either alone or in combination with existing pathway inhibitors. However, these data remain exploratory and require further functional validation.

An important future direction is to integrate this transcriptomic signature with circulating biomarkers and broader multi-omics platforms. Recent studies have demonstrated that isolated single-omics approaches often fail to capture the biological complexity of NSCLC, whereas integrative strategies that combine genomics, transcriptomics, proteomics, and liquid biopsy-derived biomarkers significantly improve diagnostic precision [5]. Chen et al. showed that combining ctDNA with RNA and protein biomarkers enhances sensitivity for early-stage cancer detection and improves patient stratification compared with mutation analysis alone [22]. Similarly, plasma-based assays incorporating exosomal RNA, circulating proteins, and methylation patterns have shown superior predictive performance compared to conventional ctDNA-only approaches.

Our current findings fit well into this emerging framework. The seven-gene panel identified here can be translated into minimally invasive platforms using circulating exosomal RNA or plasma-derived transcriptomic signatures. The use of these signatures would be particularly valuable for stage 1 NSCLC patients, in whom tissue access is often limited, and ctDNA levels are frequently below reliable detection thresholds. Moreover, integration with proteomic validation can further prioritize biologically actionable targets, particularly for genes such as *EFNA4* and *TEDC2*, where transcriptional upregulation may directly reflect functional pathway activation [24,26,33]. Such multi-layer validation would substantially increase the clinical applicability of these biomarkers and improve their potential for future diagnostic implementation.

Nonetheless, our study also has limitations. Several genes within the expression signature remain poorly characterized, necessitating functional validation via in vitro and in vivo assays. Moreover, although the RT–qPCR cohort included 20 patients, future studies should expand validation to larger and more diverse populations, including underrepresented ethnicities and patients at later stages of disease. Another important limitation of our study is the relatively small size of both the validation and clinical cohorts, which may influence the robustness and generalizability of the identified biomarker panel. Although the transcriptomic signature was consistently validated across the ENA dataset, the TCGA cohort, and our independent Hungarian patient samples, the absolute number of early-stage matched tumor–normal pairs remained limited, particularly in the RT–qPCR validation cohort. Small cohort sizes increase the risk of statistical overfitting, especially in machine learning-based feature selection approaches such as Random Forest analysis, where model performance may be artificially inflated if biological heterogeneity is underrepresented. In NSCLC, substantial variability exists across histological subtypes, smoking status, sex, ancestry, and molecular driver composition, all of which may significantly influence transcriptomic profiles. Our present cohort mainly consisted of early-stage adenocarcinoma cases; therefore, it may not fully represent the broader biological diversity of NSCLC, particularly squamous cell carcinoma or rare histological subtypes. Furthermore, prospective multicenter validation in larger, clinically heterogeneous cohorts will be necessary to determine the true diagnostic sensitivity, specificity, and reproducibility of this seven-gene panel under routine clinical conditions. Such validation will be particularly important before considering implementation in screening programs or liquid biopsy-based early detection platforms. It will also be important to assess whether the observed gene expression shifts are lung-specific or detectable in circulating compartments, such as exosomes. If validated, such expression markers can form the basis for minimally invasive, liquid biopsy-based diagnostics—especially valuable in settings where tissue sampling is limited or impossible.

## 4. Conclusions

In conclusion, our study identifies a seven-gene transcriptomic signature that effectively distinguishes early-stage NSCLC from adjacent normal tissue across three independent datasets. This gene panel holds promise not only as a diagnostic tool but also as a gateway to understanding the mechanisms underlying early lung cancer biology by combining RNA-seq-based expression profiling with machine learning-guided biomarker discovery and clinical sample validation. Our results provide a foundation for future studies aimed at supporting precision diagnostics in NSCLC. However, these tissue-based findings have to be translated into non- or minimally invasive modalities. Further research should investigate the utility of these markers in liquid biopsy formats, their specificity across different cancer types, and their potential as therapeutic targets in early intervention strategies.

## 5. Materials and Methods

### 5.1. Bioinformatic Analysis

In this study, the RNA-seq data analyzed were obtained from the European Nucleotide Archive (ENA) under Gene Expression Omnibus (GEO) accession GSE40419, corresponding to the ENA project PRJEB3132. Our inclusion criteria required matched tumor and normal samples from the same patient. We focused on early-stage samples, including 31 stage 1A and 24 stage 1B tumor samples, along with their matched adjacent normal samples. Eight tumor samples were excluded from the study cohort because they lacked accurately matched adjacent normal samples. Additionally, two sample pairs were excluded as outliers during preliminary quality control.

The initial analysis was performed in a Linux environment (Debian) using *Bioconda*-distributed tools. Quality assessment was performed with *FastQC* (version 0.12.1), followed by trimming with *Trimmomatic* (version 0.38) (SLIDINGWINDOW:4:20, MINLEN:20). After a second quality check with *FastQC*, alignment was performed with *HISAT2* (version 2.2.1) using the GRCh38 reference genome. For quantification, *FeatureCounts* (version 0.12.1), a highly efficient read summarization tool, was employed [36,37,38].

Gene expression analysis was performed in R (version 4.3.0). The packages used for visualization, data handling, and utility included *readr* (version 2.1.5), *dpylr* (version 1.1.4), *ggplot2* (version 3.5.1), and *ggrepel* (version 0.9.5). A pre-filtering step was implemented to identify and remove outliers. PCA was conducted using the *FactoMineR* (version 2.11) and *factoextra* (version 1.0.7) packages to visualize sample distances, leading to the exclusion of two additional sample pairs because the normal samples deviated significantly from the rest. Differential gene expression analysis was performed from raw counts using the *edgeR* package (version 3.42.4), identifying significant DEGs with adjusted *p*-values < 0.05 and absolute log_2_-fold changes > 1. Heatmaps were generated with the *ComplexHeatmap* package (version 2.16.0) to visualize the expression values of these genes [39,40,41,42,43,44].

For feature selection, a supervised machine learning model was built using the *caret* (version 6.0-94) and *RandomForest* (version 4.7-1.1) packages. The dataset used for the Random Forest model comprised 90 samples—45 tumors and 45 matched normals—along with normalized gene expression data. For the model, genes meeting the thresholds of |logFC| > 1 and nominal *p*-value < 0.05 were selected, resulting in 3424 genes. Since the primary goal of the model was classification, genes were filtered based on nominal *p*-values rather than adjusted *p*-values. This approach allowed the retention of additional genes that could provide useful discriminatory information, thereby improving predictive performance. Data were normalized using TPM [45].

The dataset was divided into training (80% of the data, 72 samples) and testing (20% of the data, 18 samples) sets while preserving the tumor–normal ratio. To ensure reproducibility, a fixed random seed was applied. R with 200 trees, and the mtry parameter was optimized using the square root of 3424, the number of input variables. All other settings were left at default values. Hyperparameter optimization was performed by 10-fold cross-validation. To evaluate model robustness, the entire workflow, including the data partitioning step, was repeated across 10 iterations, and the mean performance across these runs was reported.

Importance values were calculated using Mean Decrease in Accuracy, a permutation-based importance metric that is generally preferred for interpretation because it is directly tied to model prediction performance. No additional normalization or scaling was applied. Importance scores were extracted using the varImp() function with no scaling, meaning that raw importance values were used. To ensure robustness, the model employed 10-fold cross-validation, and the entire process was repeated across 10 independent iterations, where randomized data partitioning occurred each time with controlled random seeds. For each run, the decrease in accuracy was measured for each gene, and mean importance values were subsequently calculated by averaging these scores across runs. Genes were then ranked by their mean importance values, enabling the identification of those with high potential in distinguishing between classes.

For external validation, early-stage RNA-sequenced tumor–normal pairs were obtained from the TCGA–Lung Adenocarcinoma Dataset (LUAD) using the *TCGAbiolinks* package (version 2.30.0). This dataset included 29 stage 1 tumor samples with matched normal counterparts. The model was constructed using the same modeling parameters but with only the 10 highest-ranking genes, as identified by the first model’s average Mean Decrease in Accuracy (importance). In this case, the entire dataset was used for training, with an independent external TCGA dataset serving as the validation set.

Pathway enrichment analyses were also performed using the Gene Ontology (GO), Reactome, and Kyoto Encyclopedia of Genes and Genomes (KEGG) databases. Gene annotation was carried out utilizing the *bioMart* (version 2.58.0), *AnnotationDbi* (version 1.64.1), and *org.HS.eg.db* (version 3.17.0) packages. Gene set enrichment analysis (GSEA) and ORA were performed using *clusterProfiler* (version 4.8.3). The findings from the GSEA and ORA were visualized with enrichplot (version 1.20.3) [46,47,48,49].

To assess the joint discriminatory performance of the seven-gene panel, a combined ROC analysis was performed using a GLM-based approach, conceptually similar to the strategy implemented in the *combiROC* (version 0.3.4) R package. All seven genes (*CDC42EP2*, *EFNA4*, *GPM6A*, *STX11*, *TEDC2*, *THBD*, and *TMEM88*) were included as predictors in a logistic regression framework. To ensure robust estimation of model performance, a repeated random subsampling cross-validation procedure was applied. In each iteration, the dataset was randomly partitioned into training (80%) and testing (20%) subsets using stratified sampling to preserve the class distribution between tumor and normal samples. This process was repeated 20 times with a fixed random seed. For each iteration, the model was trained on the training subset and evaluated on the independent testing subset. Predicted probabilities were obtained for the testing set and used to construct ROC curves. The final ROC curve was generated by aggregating predictions across all cross-validation iterations. The analyses were performed in R. ROC analyses were conducted using the *pROC* package (version 1.19.0.1), data import was performed using *readr* (version 2.1.5), figures were generated using *svglite* (version 2.2.2), and results were exported using *openxlsx* (version 4.2.8.1) [2,3,4,5,6,7,8,9,10,11,12,13,14,15,16,17,18,19,20,21,22,23,24,25,26,27,28,29,30,31,32,33,34,35,36,37,38,39,40,41,42,43,44,45,46,47,48,49,50,51,52].

Statistical analyses of RT–qPCR data were conducted using IBM SPSS Statistics 29.0. The statistical significance of differences in expression values between normal and NSCLC samples was assessed using the independent-samples *t*-test.

The study was conducted in accordance with the Declaration of Helsinki and approved by the Scientific and Research Ethics Commission (TUKEB) of the Hungarian Medical Research Council (protocol code: BM/32453-3/2024; date of approval: 18 January 2025).

### 5.2. Cohort Characteristics

In the initial analysis, RNA-seq data from early-stage tumor samples of 55 patients were obtained from the ENA. Of these, 8 patients were excluded because no matched adjacent normal lung tissue samples were available. During PCA, an additional 2 sample pairs were removed because their corresponding normal samples clustered separately from the main group of normal tissues and were therefore considered outliers. Consequently, a total of 45 tumor samples and 45 matched adjacent normal RNA-seq samples were retained for downstream analyses, including DEG identification and Random Forest model development.

For validation, RNA-seq data from the TCGA–Lung Adenocarcinoma (LUAD) cohort were used. Specifically, 29 stage 1 tumor samples with their corresponding adjacent normal lung tissue RNA-seq samples were found and included as an independent validation dataset.

For RT–qPCR validation, a total of 24 patients, including 15 females and 9 males, diagnosed with lung cancer, were included in the study. The participants’ ages ranged from 50 to 85 years, with a mean age of 72 years. The cohort enrolled patients diagnosed with pT1a (*n* = 5), pT1b (*n* = 3), and pT1c (*n* = 16). Histopathological evaluation identified invasive solid adenocarcinoma as the predominant subtype (*n* = 9), invasive papillary adenocarcinoma (*n* = 5), invasive adenocarcinoma (*n* = 4), and mixed adenocarcinoma (*n* = 2). Less frequently observed subtypes included adenosquamous carcinoma (*n* = 1), invasive acinar adenocarcinoma (*n* = 1), mixed-type lung cancer (*n* = 1), and papillary adenocarcinoma (*n* = 1) (Table 1). However, due to extremely low RNA concentrations, four patients’ samples were excluded from further measurements and analyses. Therefore, the final RT–qPCR measurements were performed on 20 NSCLC and 20 matched adjacent healthy lung tissue samples derived from 20 patients.

### 5.3. RNA Isolation

From each patient sample, five and ten 5-micrometer slices were collected from the tumor and the corresponding normal tissue, respectively. Total RNA was isolated using the ReliaPrep RNA Cell Miniprep System Kit (Promega, Madison, WI, USA) according to the manufacturer’s instructions. The RNA concentrations were measured with a NanoDrop OneC spectrophotometer (Thermo Fisher Scientific, Waltham, MA, USA). Reverse transcription was carried out using TaqMan Reverse Transcription Reagents (Thermo Fisher Scientific, Waltham, MA, USA), according to the manufacturer’s instructions, with the following thermal profile: 25 °C for 10 min, 37 °C for 60 min, and 95 °C for 5 min. In each RT–qPCR reaction, an equal amount of cDNA was utilized.

### 5.4. RT–qPCR

For RT–qPCR evaluation, 20 patient samples were selected for further analysis. RT–qPCR reactions were conducted in a final volume of 10 µL using GoTaq qPCR Master Mix (Promega, Madison, WI, USA) with a QIAGEN Rotor-GeneQ 5-plex HRM qPCR System (Qiagen, Hilden, Germany). All RT–qPCR amplifications followed the same thermal profile: 95 °C for 7 min, 45 cycles of 95 °C for 15 s, and 60 °C for 30 s, followed by a melting curve analysis. The cycling conditions were optimized to achieve efficient amplification of the target gene while minimizing non-specific amplification. The primers, listed in Table 2, were designed using the Primer3 software (https://primer3.ut.ee/, accessed on 02-03-2025). The specificity of the primers was verified using NCBI BLAST (http://www.ncbi.nlm.nih.gov/tools/primer-blast/, version: BLAST+ 2.16.0, accessed on 02-03-2025). The primers were also tested to determine the most suitable concentrations for RT–qPCRs. All RT–qPCR reactions were performed in duplicate, and expression levels were determined based on the mean value of the duplicate measurements. For each primer pair, a no-template control (NTC) was included to detect any DNA contamination. The relative quantification method was applied in each case. In accordance with MIQE guidelines, Ct values of each sample were normalized to the geometric mean of two validated reference genes (TBP and Cyclophilin B). Relative expression levels were calculated using the ΔΔCt method, enabling accurate comparison across samples.

## 6. Patents

This work is submitted for patenting under the following accession number: European Patent Application No. 25181645.0 (June 2025).

## Figures and Tables

**Table 1:** Cohort characteristics. (**A**) Characteristics of participants included in the initial sequencing analysis obtained from the ENA dataset. (**B**) Validation cohort derived from the TCGA dataset. (**C**) Characteristics of patients included in the Hungarian population-based RT–qPCR analysis.

(**A**) **Characteristics of Participants (ENA)**			
Enrolled patients with lung cancer	*n* = 45	Smoking status	
Age interval	38–72	smoker	*n* = 19
Mean age	64	current smoker	*n* = 2
Gender		never smoked	*n* = 23
Female	*n* = 22	smoking status: NA	*n* = 1
Male	*n* = 23		
Stage			
1A	*n* = 25		
1B	*n* = 20		
(**B**) **Characteristics of participants (TCGA)**			
Enrolled patients with lung cancer	*n* = 29	Histological subtype	
Age interval	51–86	Adenocarcinoma, NOS	*n* = 23
Mean age	67	Bronchiolo-alveolar carcinoma, non-mucinous	*n* = 1
Gender		Mucinous adenocarcinoma	*n* = 1
Female	*n* = 18	Adenocarcinoma with mixed subtypes	*n* = 1
Male	*n* = 11	Papillary adenocarcinoma	*n* = 1
(**C**) **Characteristics of participants (RT–qPCR)**			
Enrolled patients with lung cancer	*n* = 24	Histological subtype	
Age interval	50–85	Invasive solid adenocarcinoma	*n* = 9
Mean age	72	Invasive papillary adenocarcinoma	*n* = 5
Gender		Invasive adenocarcinoma	*n* = 4
Female	*n* = 15	Mixed adenocarcinoma	*n* = 2
Male	*n* = 9	Adenosquamous carcinoma	*n* = 1
Tumor size (T)		Invasive acinar adenocarcinoma	*n* = 1
pT1a	*n* = 5	Mixed-type lung cancer	*n* = 1
pT1b	*n* = 3	Papillary adenocarcinoma	*n* = 1
pT1c	*n* = 16		

**Table 2:** Primer sequences for the genes of interest and the housekeeping genes used in RT–qPCR.

Gene	Forward Primer (5’–3’)	Reverse Primer (5’–3’)
*TEDC2*	CTCAAGGAGAAGGGGCACC	TGGTCTGTGTGGAACTGAGC
*TMEM88*	CACTCTCAGTTCCTGCGCTC	CGATAAAGGGCTCGGCTGTA
*GPM6A*	TGCGAATCTACTGAGCTGAAC	CCAGTTGGCAGACAGAACCA
*STX11*	AAGTGGGACGTGTTTTCCGA	CTCTCGATCTCGTTGAGGGC
*THBD*	CCTAATGACAGTGCGCTCCT	CTGGTGTTGTTGTCTCCCGT
*CDC42EP2*	TATCTGAAGCGTGGCAGTCG	ATGAATGGTGTGGCGGAAGT
*EFNA4*	TTACTACTACATCTCGGTGCCC	GAAGACGAAGAATCAGAAGCAG
*CYCB*	CTTCCCCGATGAGAACTTCAAACT	CACCTCCATGCCCTCTAGAACTTT
*TBP*	ACTCCACTGTATCCCTCCCC	TATATTCGGCGTTTCGGGCA

## Data Availability

The original contributions presented in this study are included in the article/Supplementary Materials. Further inquiries can be directed to the corresponding author(s).

## Supplementary Materials

The following supporting information can be downloaded at: https://www.mdpi.com/article/10.3390/ijms27104282/s1.

## Abbreviations

The following abbreviations are used in this manuscript:

ALK	anaplastic lymphoma kinase
BRAF	B-Raf proto-oncogene, serine/threonine kinase
CDC42EP2	CDC42 effector protein 2
ctDNA	circulating tumor DNA
EFNA4	ephrin A4
EGFR	epidermal growth factor receptor
ENA	European Nucleotide Archive
GO	Gene Ontology
GPM6A	glycoprotein M6A
GSEA	Gene set enrichment analysis
HER2	human epidermal growth factor receptor 2
KEGG	Kyoto Encyclopedia of Genes and Genomes
KRAS	Kirsten rat sarcoma viral oncogene homolog
NGS	next-generation sequencing
NSCLC	Non-small cell lung cancer
ORA	over-representation analysis
PCA	Principal Component Analysis
PCR	polymerase chain reaction
PD-L1	programmed death-ligand 1
RNA-seq ROC	RNA sequencing Receiver Operating Characteristic
ROS1	ROS proto-oncogene 1, receptor tyrosine kinase
RT–qPCR	Reverse Transcription Quantitative Polymerase Chain Reaction
STX11	syntaxin 11
TCGA	The Cancer Genome Atlas
TEDC2	tubulin epsilon and delta complex 2
THBD	thrombomodulin
TMEM88	transmembrane protein 88
TPM	transcripts per million
