# Supplementary material for: Machine Learning-Based Identification of Biomarkers for Early-Stage Non-Small Cell Lung Cancer Through Gene Expression Analysis
Source: Int J Mol Sci. 2026 May 11;27(10):4282. doi: 10.3390/ijms27104282 (PMC13207127; doi:10.3390/ijms27104282)

Supplementary Figure S1. Pathway Analyses of the DEGs from the ENA samples.

(**A**) Category net plot of enriched KEGG pathways identified by GSEA. (**B, C**) Results of the GSEA using the GO database. (**D, E**) Dot plot and category net plot of the GSEA based on the Reactome database. Results highlighting activation in cell cycle and DNA repair pathway, consistent with cancer-related processes. (**F**) The results of ORA using the GO database visualized on a category net plot. (**G, H**) ORA results based on the KEGG database, showing enrichment of neuroactive ligand–receptor interaction, calcium signaling, and cell adhesion pathways.

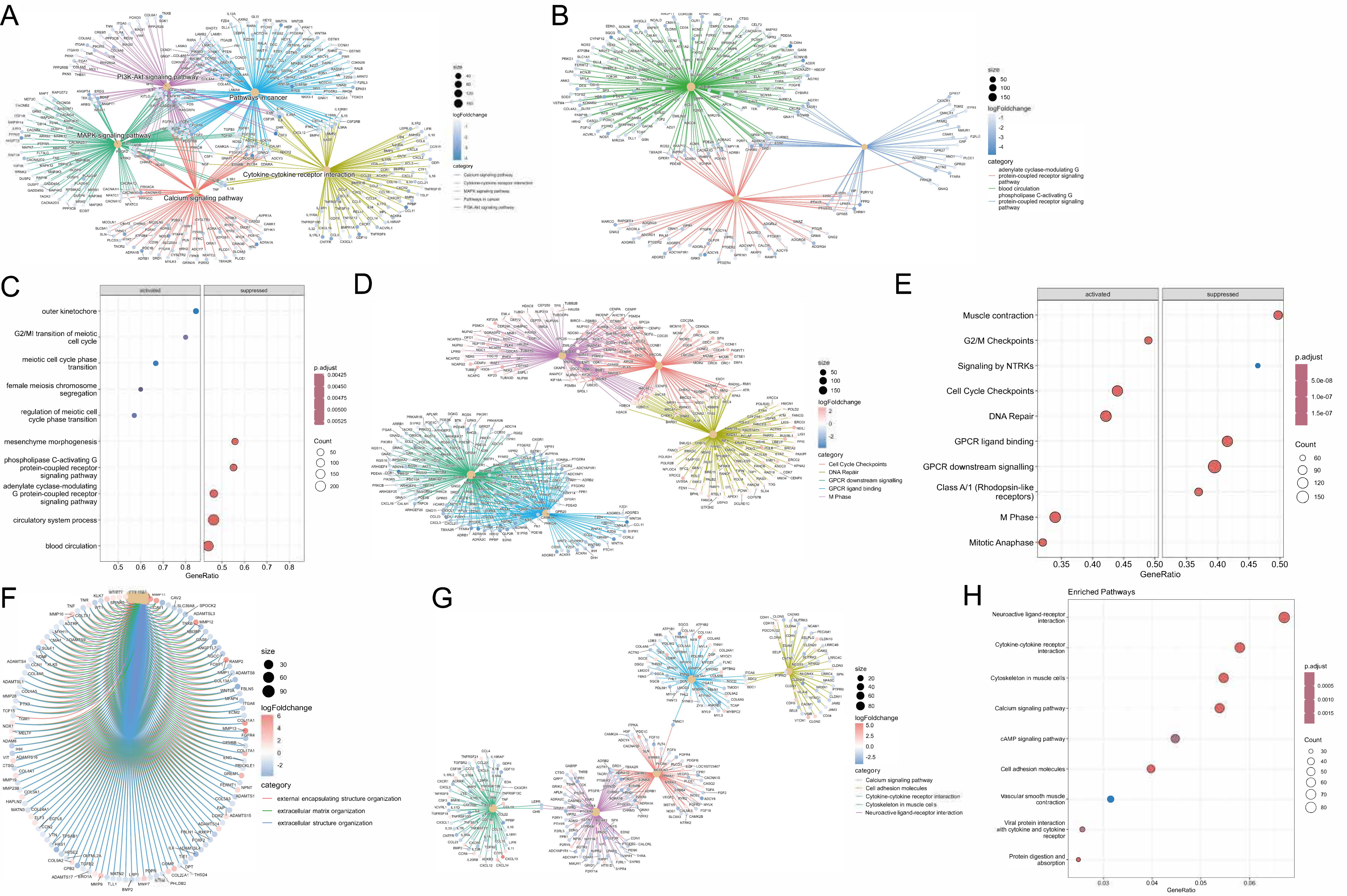

Supplementary Figure S2. Pathway Analyses of the DEGs from the TCGA samples.

(**A**) Category net plot of enriched KEGG pathways identified by GSEA. (**B, C**) Results of the GSEA using the GO database. (**D, E**) Dot plot and category net plot of the GSEA based on the Reactome database, with the most significantly affected pathways related to cell cycle checkpoints and mitotic phase processes. (**F**) The results of ORA using the GO database visualized on a category net plot. (**G, H**) Enriched terms of the ORA based on KEGG database shown on these plots.

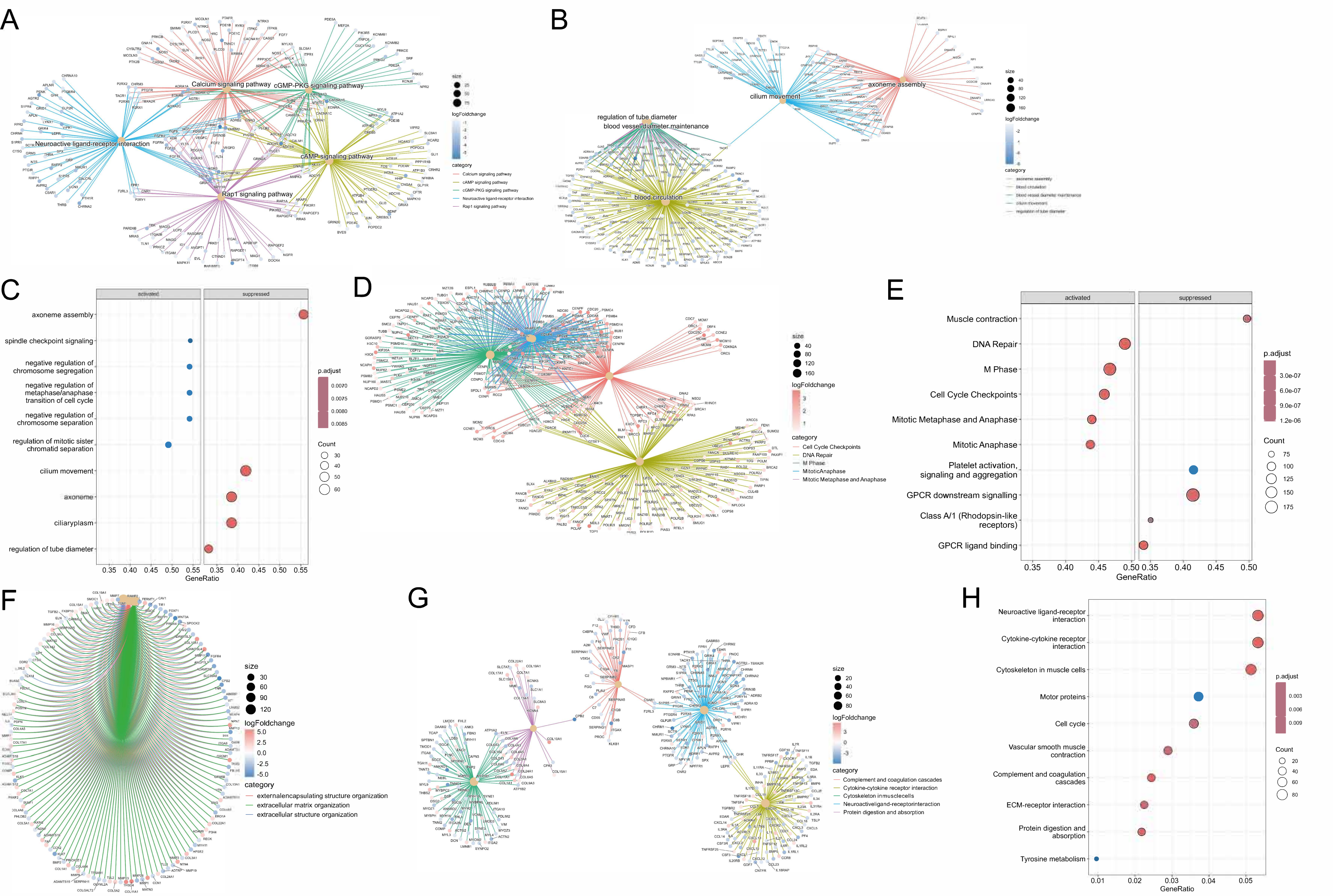

Supplement: Supplementary file 1 [file ijms-27-04282-s001.zip › ijms-4267039-supplementary.pdf]
